# Retinal Thickness in Essential Tremor and Early Parkinson Disease: Exploring Diagnostic Insights

**DOI:** 10.1097/WNO.0000000000001959

**Published:** 2023-07-31

**Authors:** Claudio Terravecchia, Giovanni Mostile, Clara G. Chisari, Cristina Rascunà, Roberta Terranova, Calogero E. Cicero, Loretta Giuliano, Giulia Donzuso, Giorgia Sciacca, Antonina Luca, Pierre-Marie Preux, Joseph Jankovic, Mario Zappia, Alessandra Nicoletti

**Affiliations:** Department “G.F. Ingrassia” (CT, GM, CGC, CR, RT, CEC, LG, GD, GS, AL, MZ, AN), Section of Neurosciences, University of Catania, Catania, Italy; Oasi Research Institute—IRCCS (GM), Troina, Italy; INSERM (P-MP), University of Limoges, CHU Limoges, IRD, U1094 Tropical Neuroepidemiology, Institute of Epidemiology and Tropical Neurology, GEIST, Limoges, France; and Parkinson's Disease Center and Movement Disorders Clinic (JJ), Department of Neurology, Baylor College of Medicine, Houston, Texas.

## Abstract

**Background:**

Essential tremor (ET) represents a heterogeneous condition which may overlap with Parkinson disease (PD) even at early stages, by sharing some subtle clinical aspects. Longstanding ET demonstrated also higher risk of developing PD, especially with a Tremor-dominant (TD-PD) phenotype. Therefore, differential diagnosis between ET and early PD could be quite challenging. Optical coherence tomography (OCT) has been recognized as a reliable tool to assess the retina as a proxy of neurodegeneration. We aimed to explore the possible role of retinal assessment in differential diagnosis between ET and early PD.

**Methods:**

Macular layers and peripapillary retinal nerve fiber layer (RNFL) thickness among ET, early PD, and healthy controls (HCs) were assessed using OCT.

**Results:**

Forty-two eyes from 23 ET, 41 eyes from 21 early PD, and 33 eyes from 17 HCs were analyzed. Macular RNFL, ganglion cell layer, inner plexiform layer, and inner nuclear layer were thinner in PD as compared with ET and even more in HCs. Differences between ET and PD were more evident when considering the TD-PD subgroup, especially for RNFL. Among ET patients, thickness of the inner macular layers showed negative linear relationship with both age at onset and disease duration. Peripapillary temporal quadrant thinning was found in ET compared with HCs.

**Conclusions:**

Macular inner retina was thinner in patients with ET and early PD compared with HCs. These findings suggest that the retinal assessment may have a utility in the differential diagnosis between ET and PD.

In the past few decades, a growing body of clinical–instrumental evidence has changed the notion of essential tremor (ET) from a “benign,” monosymptomatic disease to a progressive neurodegenerative condition characterized by a broad phenotypical heterogeneity.^1^ To better characterize this heterogeneity, in 2018, a new Consensus Statement on the Classification of Tremors has been proposed, introducing the distinction between “ET” and “ET-plus.”^2^ The latter represents a separate nosological entity defined as a “*tremor with the characteristics of essential tremor, with additional neurological signs of uncertain clinical significance*” that “*do not suffice to make an additional syndrome classification or diagnosis*.” Nevertheless, this newly introduced term raised several concerns and controversies mainly due to uncertainties in the clinical definitions of required additional “*soft signs*” for the diagnosis of ET-plus,^1^ which include questionable dystonic posturing, impaired tandem gait, memory impairment, or rest tremor.^2^ Patients with longstanding ET may develop such ancillary “*soft signs*,” including rest tremor, leading to a clinical overlap with early Parkinson disease (PD).^3^ Considering that a subset of ET subjects may present a high risk of developing PD (the so-called “ET-PD syndrome”), these clinical features raise the challenging question of whether they represent the manifestation of an early-PD or an ET subtype. Moreover, studies of large ET families found an association between ET and PD, particularly the tremor-dominant PD (TD-PD) phenotype.^3^

Functional neuroimaging has been used to obtain diagnostic and prognostic information in PD.^4,5^ Although disease-specific diagnostic biomarkers are not available to reliably differentiate between ET and PD, abnormalities in retinal thickness have emerged as a possible marker of several neurological disorders, including PD and ET.^6–12^ In addition to retinal thickness abnormalities, we also previously demonstrated that microvascular changes in the retina may occur even in early stages of PD.^11^ To date, few studies evaluated retinal abnormalities in ET and only 3^7,9,10^ investigated the possible role of optical coherence tomography (OCT) in differentiating ET from PD but reporting conflicting results. Methodological differences limit the comparison of findings across the studies.^7–10^ Moreover, to date, no evidence has been provided regarding retinal differences between ET and early PD or among PD phenotypes.

In this study, we aimed to assess retinal abnormalities in long-lasting ET as compared with early PD through a standardized OCT protocol, focusing on different PD phenotypes to explore the possible differential diagnostic role of retinal assessment in ET.

## METHODS

### Study Population

Three groups of subjects were enrolled: ET patients, PD patients, and healthy controls (HCs). The selection criteria for PD and HCs were described in our previous study.^11^ In this study, patients with ET, recruited from the “Parkinson's Disease and Movement Disorders Centre” of the University of Catania, Italy, fulfilled the diagnostic criteria for “classic ET” according to Consensus Statement on Tremor of the Movement Disorder Society (definite/probable ET based on TRIG classification).^13^ A disease duration minimum cut-off of 3 years was adopted as inclusion criterion for ET study subjects. Patients with early PD were enrolled in this study if they fulfilled the MDS-PD diagnostic criteria for clinically established or clinically probable PD.^14^ They were divided into either TD-PD, postural instability/gait difficulty-PD (PIGD-PD), or indeterminate-PD (Ind-PD) according to established criteria.^15^ A symptom duration longer than 5 years was an exclusion criterion for the early PD group. A group of HCs, selected among patients' caregivers attending our clinic without any evidence of neurodegenerative disease, was also enrolled in the study. All study subjects underwent a complete standardized neurological examination performed by a neurologist expert in movement disorders. In patients, motor assessment was performed using Tremor Research Group—Essential Tremor Rating Assessment Scale (TETRAS) and Unified Parkinson's Disease Rating Scale part-III (UPDRS-III); global cognitive status was assessed by Mini-Mental State Examination (MMSE). All subjects underwent OCT analysis. Exclusion criteria included history of ocular trauma, previous ocular surgery that could impair the visual pathway or macular morphology; concurrent ocular diseases; increased intraocular pressure; media opacifications; and systemic conditions that could impair visual system, such as diabetes mellitus, uncontrolled hypertension or hypotension, cardiovascular diseases, and any other neurological disease. Study protocol was approved by the local ethics committee. Written informed consents were obtained from study subjects.

### High-Definition Spectral-Domain Optical Coherence Tomography Imaging Protocol

Macular retinal thickness and peripapillary retinal nerve fiber layer (RNFL) thickness were assessed using the Spectral Domain Cirrus HD OCT model 5000 (Carl Zeiss Meditec, Inc, Jena, Germany). Patients were evaluated in the same day of neurological examination in a shaded room light without previous pharmacological pupil dilation by a single examiner (C.G.C.). The same device was used for all the study subjects. To examine the macula, the Macular Cube 512 × 128 protocol was applied. It uses a raster scan mode that scans a 6 × 6-mm macular area into 512 × 128 (length by width) points. Layer segmentation of the OCT data was performed using a previously developed and validated algorithm. The algorithm had 3 stages: preprocessing, pixel classification, and graph-based multilayer segmentation. In addition, estimates of the inner and outer retinal boundaries (inner limiting membrane and Bruch membrane) were used to restrict the region of interest for the algorithm, as well as to flatten the data to the Bruch membrane boundary. Constraints were used to limit the minimum and maximum distance between each boundary and to limit the smoothness of the final segmentation. Moving from the inside to the outside, the macula was defined by the following layers: RNFL, ganglion cell layer (GCL), inner plexiform layer (IPL), inner nuclear layer (INL), outer plexiform layer, outer nuclear layer (ONL), and retinal pigment epithelium.

Peripapillary RNFL thickness was acquired with the Optic Disc Cube 200 × 200 protocol that images the optic disc in a 6 × 6 mm region. The average peripapillary RNFL (pRNFL) thickness and the pRNFL thickness along the superior, temporal, inferior, and nasal sectors were also assessed. For each subject, retinal images of both left and right eye were acquired and analyzed. Low-quality measurements were excluded from the analysis. The OCT protocol was reported according to APOSTEL 2.0 recommendations.^16^

### Statistical Analysis

Data were analyzed using STATA 12.1 software packages. Quantitative variables were described using mean and SD. The difference between proportions was assessed by the chi-squared test. The difference between means was evaluated by the *t* test and analysis of variance (ANOVA) implemented by the Bonferroni post hoc test. OCT data were analyzed using 2-factors split-plot ANOVA, with the patient group as the main plot factor and eye as subplot factor, to evaluate any significant differences between right and left eyes and their possible effects on differences among groups.^17^ In case of a not-normal distribution, appropriate nonparametric tests were performed.

To evaluate the possible association between ET and thickness of each retinal layers as compared with PD and HCs, an unconditional logistic regression analysis was performed by considering the presence of ET as the outcome variable. The odds ratios (ORs) with 95% confidence intervals (CIs) and *P*-value (2-tailed test, a = 0.05) were calculated. For each retinal layer, the multivariate model was constructed considering age and sex as a priori confounders. Parameters associated with the outcome at the univariate analysis with a threshold of *P* = 0.10 were included in the model. Pearson analysis was performed to evaluate possible correlation between retinal thickness and clinical features in the ET group. Multiple linear regression analysis was performed to investigate the independent effect of each scalar variable after adjusting for possible confounding variables.

## RESULTS

### Descriptive Analysis

Twenty-three ET patients (14 men, 60.9%; age: 72.3 ± 6.5 years), 21 PD patients (12 men, 57.1%; age: 61.5 ± 6.5 years), and 17 HCs (9 men, 52.9%; age: 65.1 ± 10.7 years) were enrolled in the study. PD patients had a short disease duration (2.3 ± 1.2 years) and a mean UPDRS-III score of 25.0 ± 6.9. Eight of them were receiving dopaminergic treatment (levodopa equivalent daily dosage: 127.4 ± 142.7 mg). Of the 21 PD patients, 8 were classified as TD-PD, 10 PIGD-PD, and 3 Ind-PD. Of the 23 ET subjects which were enrolled based on adopted diagnostic criteria, 9 (39.1%) presented resting tremor of the limbs; thus, they could be reclassified as ET-plus based on the current proposed classification.^2^ ET patients were significantly older compared with patients in the other groups, and a statistically significant different prevalence of hypertension was found among PD, ET, and HCs as well as among PD phenotypes (Table 1). Moreover, statistically significant differences in clinically-assessed tremor pattern were found between ET and early PD (Table 1).

**TABLE 1.** Clinical characteristics and instrumental parameters among groups

	PD N = 21 (41 Eyes)	ET N = 23 (42 Eyes)	HC N = 17 (33 Eyes)	ANOVA *P*	PD vs ET *P*	ET vs HC *P*	PD vs HC *P*
Clinical characteristics							
Sex (M)	12 (57.1%)	14 (60.9%)	9 (52.9%)	0.886	1.000	1.000	1.000
Age	61.5 ± 6.5	72.3 ± 6.5	65.1 ± 10.7	**<0.001**	**<0.001**	**0.018**	0.534
Age at onset	59.3 ± 7.0	58.8 ± 18.4	/	0.898		/	/
Disease duration (yr)	2.3 ± 1.2	13.6 ± 15.4	/	**0.002**		/	/
MMSE	27.0 ± 2.7	28.3 ± 2.0	27.0 ± 2.3	0.184	0.229	0.320	1.000
Hypertension (%)	13 (61.9)	18 (78.3)	4 (23.5)	**0.001**	0.394	**0.001**	0.084
UPDRS-III	25.0 ± 6.9	14.9 ± 10.0	2.7 ± 1.8	**<0.001**	**<0.001**	**<0.001**	**<0.001**
TETRAS-performance total	/	21.3 ± 9.1	/	**/**	/	/	/
Tremor characteristics							
Head (%)	3 (14.3)	14 (60.9)	/	/	**0.002**	/	/
Voice (%)	0	14 (60.9)	/	/	**<0.001**	/	/
Upper limbs							
Rest tremor (%)	13 (61.9)	9 (39.1)	/	/	0.131	/	/
Postural tremor (%)	12 (57.1)	20 (87.0)	/	/	**0.027**	/	/
Kinetic tremor (%)	7 (33.3)	23 (100.0)	/	/	**<0.001**	/	/
Lower limbs							
Rest tremor (%)	6 (28.6)	1 (4.3)	/	/	**0.028**	/	/
Postural tremor (%)	0	5 (21.7)	/	/	**0.023**	/	/
Kinetic tremor (%)	0	5 (21.7)	/	/	**0.023**	/	/
OCT parameters							
RNFL (µm)	13.4 ± 1.9	15.0 ± 1.6	17.8 ± 2.2	**<0.001**	**<0.001**	**<0.001**	**<0.001**
GCL (µm)	16.1 ± 3.2	17.6 ± 3.4	21.4 ± 2.2	**<0.001**	0.095	**<0.001**	**<0.001**
IPL (µm)	21.4 ± 2.9	22.1 ± 3.3	24.2 ± 2.1	**<0.001**	0.799	**0.005**	**<0.001**
INL (µm)	20.7 ± 5.5	23.5 ± 5.1	28.2 ± 4.5	**<0.001**	**0.043**	**<0.001**	**<0.001**
OPL (µm)	28.5 ± 6.3	27.9 ± 5.1	31.2 ± 4.8	**0.030**	1.000	**0.035**	0.119
ONL (µm)	86.1 ± 12.6	87.3 ± 9.9	97.7 ± 7.7	**<0.001**	1.000	**<0.001**	**<0.001**
RPE (µm)	15.6 ± 1.6	15.7 ± 1.6	16.3 ± 1.7	0.168	1.000	0.388	0.227
pRNFL (µm)	92.8 ± 9.4	95.5 ± 10.0	101.1 ± 7.9	**<0.001**	0.571	**0.029**	**<0.001**
pRNFL superior (µm)	112.8 ± 16.5	114.0 ± 11.2	120.6 ± 9.6	**0.026**	1.000	0.089	**0.032**
pRNFL temporal (µm)	70.5 ± 9.8	72.7 ± 7.4	78.3 ± 7.8	**<0.001**	0.677	**0.016**	**<0.001**
pRNFL inferior (µm)	120.2 ± 15.0	115.5 ± 11.5	119.4 ± 9.2	0.189	0.259	0.533	1.000
pRNFL nasal (µm)	73.0 ±10.2	75.3 ± 9.4	80.9 ± 8.2	**0.002**	0.779	**0.036**	**0.001**

In bold: *P* < 0.05.

ANOVA, analysis of variance; ET, essential tremor; GCL, ganglion cell layer; HC, healthy controls; INL, inner nuclear layer; IPL, inner plexiform layer; MMSE, mini mental state examination; ONL, outer nuclear layer; OPL, outer plexiform layer; PD, Parkinson disease; pRNFL, peripapillary RNFL; RNFL, retinal nerve fiber layer; RPE, retinal pigment epithelium; TETRAS, The Essential Tremor Rating Assessment Scale; UPDRS-III, Unified Parkinson's Disease Rating Scale—Part III.

### Optical Coherence Tomography Analysis–Comparison of Macular Retinal Layers and Peripapillary Retinal Nerve Fiber Layer Thickness

A total of 42 eyes from 23 ET, 41 eyes from 21 PD, and 33 eyes from 17 HCs were analyzed. Four eyes from ET patients, 1 eye from PD, and 1 eye from HCs were excluded because of the poor OCT imaging quality. No significant differences in retinal layers' thickness were found between right and left eyes in ET, PD, and HCs groups. Group × eye interaction analysis suggested that significant differences between groups were similar for right and left eyes (Table 2). Among ET patients, we found a negative correlation between age at onset and GCL (r = −0.47, 0.002), IPL (r = −0.56, *P* < 0.001), INL (r = −0.50, *P* < 0.001), and OPL (r = −0.39, *P* = 0.011), whereas a positive correlation was found between disease duration and GCL (r = 0.43, *P* = 0.005), IPL (r = 0.44, *P* = 0.003), INL (r = 0.38, *P* = 0.012), and OPL (r = 0.33, *P* = 0.033). At multivariate analysis, significant multiple linear regression models considering both age at onset and disease duration as independent variables demonstrated a negative linear relationship between such variables and RNFL (coefficients: −0.10 [*P* = 0.012] and −0.11 [*P* = 0.022], respectively), IPL (coefficients: −0.23 [*P* = 0.039] and −0.17 [*P* = 0.039], respectively), and INL (coefficients: −0.35 [*P* = 0.003] and −0.27 [*P* = 0.044], respectively).

**TABLE 2.** Two-factors split-plot analysis of variance analysis

							Two-Factors Split-Plot ANOVA			
	PD N = 21 (41 eyes)		ET N = 23 (42 eyes)		HC N = 17 (33 eyes)		Model F (*P*)	Groups F (*P*)	Eye F (*P*)	Groups × Eye F (*P*)
	Right Eye	Left Eye	Right Eye	Left Eye	Right Eye	Left Eye				
RNFL (µm)	13.6 ± 2.2	13.2 ± 1.6	15.0 ± 1.6	15.0 ± 1.7	17.9 ± 1.8	17.6 ± 2.5	**19.76 (<0.001)**	**48.98 (< 0.001)**	0.54 (0.463)	0.11 (0.9)
GCL (µm)	15.8 ± 3.4	16.4 ± 2.9	17.5 ± 3.7	17.6 ± 3.2	21.5 ± 2.3	21.4 ± 2.2	**11.48 (<0.001)**	**28.41 (<0.001)**	0.17 (0.684)	0.11 (0.9)
IPL (µm)	20.8 ± 3.5	21.9 ± 2.1	21.5 ± 3.2	22.7 ± 3.3	24.8 ± 1.6	23.6 ± 2.4	**4.86 (<0.001)**	**9.48 (< 0.001)**	0.5 (0.479)	2.04 (0.135)
INL (µm)	19.9 ± 6.5	21.6 ± 4.2	23.5 ± 5.4	23.5 ± 5.0	27.3 ± 4.7	29.1 ± 4.4	**8.22 (<0.001)**	**19.5 (<0.001)**	1.51 (0.222)	0.39 (0.675)
OPL (µm)	26.5 ± 6.8	30.5 ± 5.0	27.5 ± 5.9	28.4 ± 4.4	30.7 ± 3.2	31.7 ± 6.2	**2.8 (0.02)**	**3.71 (0.027)**	4.01 (0.05)	1.13 (0.327)
ONL (µm)	85.8 ± 15.6	86.4 ± 8.7	87.8 ± 10.3	86.9 ± 9.6	97.5 ± 7.0	97.9 ± 8.6	**5.16 (<0.001)**	**12.84 (<0.001)**	0.001 (0.99)	0.05 (0.947)
RPE (µm)	15.4 ± 1.9	15.7 ± 1.3	15.4 ± 1.6	16.0 ± 1.5	16.3 ± 1.8	16.2 ± 1.7	1.09 (0.368)	1.77 (0.174)	0.94 (0.334)	0.37 (0.688)
pRNFL (µm)	91.8 ± 10.4	93.8 ± 8.4	94.9 ± 10.3	96.0 ± 9.8	100.0 ± 6.8	102.3 ± 9.1	**3.21 (0.01)**	**7.48 (<0.001)**	1.07 (0.303)	0.05 (0.951)
pRNFL superior (µm)	109.8 ± 18.4	115.9 ± 14.0	114.0 ± 11.5	114.0 ± 11.2	119.6 ± 7.5	121.7 ± 11.5	1.99 (0.08)	**3.74 (0.027)**	1.27 (0.262**)**	0.57 (0.565)
pRNFL temporal (µm)	70.1 ± 9.6	70.8 ± 10.3	73.0 ± 7.3	72.4 ± 7.6	78.6 ± 8.7	77.9 ± 7.1	**3.2 (0.01)**	**7.88 (<0.001)**	0.01 (0.91)	0.07 (0.929)
pRNFL inferior (µm)	119.4 ± 15.3	121.0 ± 15.1	115.8 ± 11.7	115.2 ± 11.7	119.4 ± 8.0	119.3 ± 10.6	0.7 (0.627)	1.66 (0.195)	0.02 (0.901)	0.08 (0.919)
pRNFL nasal (µm)	73.9 ±11.1	72.0 ± 9.3	75.5 ± 9.0	75.1 ± 10.1	82.4 ± 6.0	79.3 ± 10.0	**2.9 (0.017)**	**6.55 (0.002)**	1.01 (0.317)	0.19 (0.828)

In bold: *P* < 0.05.

ANOVA, analysis of variance; ET, essential tremor; GCL, ganglion cell layer; HC, healthy controls; INL, inner nuclear layer; IPL, inner plexiform layer; ONL, outer nuclear layer; OPL, outer plexiform layer; PD, Parkinson disease; pRNFL, peripapillary RNFL; RNFL, retinal nerve fiber layer; RPE, retinal pigment epithelium.

Macular RNFL, GCL, IPL, INL, OPL, and ONL thickness were statistically different among the 3 study groups. A progressive thinning from HCs to PD with intermediate values in ET patients was found (Table 1). These findings were partially confirmed by multivariate logistic regression analysis, adjusting by age, sex, and hypertension. In particular, macular RNFL, GCL, and INL were thinner in PD as compared with ET and even more with HCs. Moreover, a lower ONL thickness was found in ET compared with HCs and IPL thinning was found to be greater in PD than in ET (Table 3). Concerning PD phenotypes, no significant differences in retinal layers were found between TD-PD and PIGD-PD. The differences found between ET and PD were even more evident when ET was compared with the TD-PD group, especially in RNFL (adjusted-odds ratio [*P*-value]: 3.17 [<0.001] and 4.61 [0.002], respectively).

**TABLE 3.** Multivariate analysis of differences in instrumental parameters among groups

	ET vs HC		ET vs PD	
OCT Parameters	AdjOR* (95% CI)	*P*	AdjOR* (95% CI)	*P*
RNFL (µm)	0.51 (0.33–0.78)	**0.002**	3.17 (1.74–5.77)	**<0.001**
GCL (µm)	0.49 (0.33–0.74)	**0.001**	1.30 (1.04–1.60)	**0.017**
IPL (µm)	0.82 (0.63–1.07)	0.153	1.40 (1.08–1.82)	**0.010**
INL (µm)	0.84 (0.72–0.98)	**0.031**	1.15 (1.00–1.31)	**0.042**
OPL (µm)	0.88 (0.75–1.02)	0.104	1.00 (0.91–1.10)	0.998
ONL (µm)	0.92 (0.85–0.99)	**0.038**	1.03 (0.98–1.08)	0.284
RPE (µm)	0.91 (0.61–1.36)	0.641	1.06 (0.72–1.54)	0.771
pRNFL (µm)	0.96 (0.87–1.03)	0.261	1.05 (0.99–1.11)	0.128
pRNFL superior (µm)	0.95 (0.89–1.02)	0.206	1.02 (0.98–1.06)	0.338
pRNFL temporal (µm)	0.88 (0.81–0.97)	**0.007**	1.03 (0.96–1.10)	0.441
pRNFL inferior (µm)	0.99 (0.93–1.05)	0.736	1.00 (0.95–1.04)	0.820
pRNFL nasal (µm)	0.96 (0.89–1.03)	0.285	1.06 (1.00–1.13)	0.052

In bold: *P* < 0.05.

* Adjusted OR by age, sex, and hypertension.

ET, essential tremor; GCL, ganglion cell layer; HC, healthy controls; INL, inner nuclear layer; IPL, inner plexiform layer; ONL, outer nuclear layer; OPL, outer plexiform layer; PD, Parkinson disease; pRNFL, peripapillary RNFL; RNFL, retinal nerve fiber layer; RPE, retinal pigment epithelium.

Regarding peripapillary RNFL, significant differences were found across the study groups considering the whole peripapillary region as well as its quadrants, except for the inferior (Table 1). Only the thinning of temporal quadrant in ET as compared with HCs was confirmed after adjusting by age, sex, and hypertension (Table 3).

## CONCLUSIONS

In this study, we found significant differences in macular thinning of RNFL, GCL, IPL, and INL among early PD, ET, and HC, with ET showing intermediate values between the other 2 groups. The differences between ET and PD were particularly pronounced when considering the TD-PD subgroup, particularly for RNFL. Moreover, among patients with ET an independent negative linear relationship between both age at onset and disease duration and RNFL, IPL, and INL thickness was found. As far as we know, this is the first study comparing retinal thickness in ET and early PD using a complete segmentation of all retinal layers and a stratified analysis for different PD phenotypes.

To date, only one study previously described the retinal pattern in ET and HC using segmentation analysis.^8^ However, it only focused on the inner retinal layers (RNFL, GCL, and IPL), whereas the entire retina (both internal and external) was segmented in our study. Consistent with our results, a thinning of RNFL, GCL, and IPL in ET compared with HC was documented.^8^ As far as we know, only 3 other studies compared the retinal pattern in PD, ET, and HCs, finding retinal impairment in PD as compared with ET.^7,9,10^ Nevertheless, among them, only in one recent study an inner retinal segmentation analysis was performed.^10^ In particular, Cubo et al^7^ conducted a first pilot study showing global foveal thinning in patients with PD compared with ET and HCs. On the other hand, Tugcu et al^9^ described lower parafoveal macular thickness and thinner peripapillary RNFL in PD with respect to ET patients. Finally, in line with our results, Satue et al^10^ showed a macular RNFL thinning in PD compared with ET as well as a reduced macular GCL thickness in ET in respect to HCs.

Regarding the peripapillary region, we found no differences between ET and PD. However, a thinning in the temporal sector was found in ET as compared with HCs. Our results differ from previous reports showing thinner RNFL in the nasal and inferonasal peripapillary quadrant in ET.^8,10^ Interestingly, on the other hand, an involvement of the temporal region in neurodegenerative conditions such as PD has been previously described.^11,18^

Taken together, our results provide an evidence of a patterned retinal degeneration involving inner macular layers with a progressively increasing severity in ET and early stages of PD.

Because an impairment of the inner retina has been proposed as a proxy of neurodegeneration,^6^ our findings may support possible neurodegenerative processes involved in ET pathophysiology. On this ground, the present results are in line with our previous data-based multimodal study demonstrating greater retinal impairment in patients with poorer performance on motility and cognition.^19^ Interestingly, in the ET group, a greater inner retinal impairment was independently associated with both longer disease duration and later disease onset. Literature data suggest a worst disease progression rate in late onset as compared with early-onset ET patients.^20^ On this ground, considering the retinal thickness a proxy of neurodegeneration, it is possible to hypothesize a greater neurodegenerative burden among late-onset ET.

Some limitation of our study should be pointed out. The sample size in all 3 groups is relatively small. Moreover, because of the cross-sectional design of this study, we cannot rule out that at least some of the ET patients with major retinal impairment would subsequently develop PD.

Despite these limitations, the present investigation provides a comprehensive analysis of retinal segmentation in ET in comparison with early PD and HCs, possibly suggesting a continuum between these explored conditions. We conclude that OCT assessment of the inner retina may facilitate differential diagnosis between ET and early PD. Longitudinal investigation is needed to assess the predictive value of OCT in identifying ET patients with high risk of subsequently developing PD.

STATEMENT OF AUTHORSHIP

Conception and design: C. Terravecchia, G. Mostile, M. Zappia, A. Nicoletti; Acquisition of data: C. Terravecchia, C. G. Chisari, C. Rascunà, R. Terranova; Analysis and interpretation of data: C. Terravecchia, G. Mostile, C. G. Chisari, C. E Cicero, A. Luca, A. Nicoletti. Drafting the manuscript: C. Terravecchia, G. Mostile; Revising the manuscript for intellectual content: C. G. Chisari, C. Rascunà, R. Terranova, C. E. Cicero, L. Giuliano, G. Donzuso, G. Sciacca, A. Luca, P.-M. Preux, J. Jankovic, M. Zappia, A. Nicoletti. Final approval of the completed manuscript: C. Terravecchia, G. Mostile, C. G. Chisari, C. Rascunà, R. Terranova, C. E. Cicero, L. Giuliano, G. Donzuso, G. Sciacca, A. Luca, P.-M. Preux, J. Jankovic, M. Zappia, A. Nicoletti.
